# A stereotaxic surgical protocol for posterior and total corpus callosotomy in adult Wistar rats using the WIN-PTZ kindling model

**DOI:** 10.1016/j.mex.2026.104123

**Published:** 2026-08-27

**Authors:** Vitória Bernal Cavalcanti, Larissa Matsusako, Luciana Canabarro, Fábio Nakabashi, Filipe Feitosa, Willy Marcus Gomes França, Paulo Henrique Pires de Aguiar

**Affiliations:** Faculdade de Ciências Médicas e da Saúde, Pontifícia Universidade Católica de São Paulo (PUC-SP), Sorocaba, Brazil

**Keywords:** Corpus callosotomy, Posterior callosotomy, Stereotaxic surgery, Wistar rat, Ptz kindling, Racine scale, Interhemispheric disconnection, Epilepsy model

## Abstract

Corpus callosotomy is an established palliative surgical procedure for drug-resistant generalized epilepsy. Although total callosotomy (TC) is the conventional approach, posterior callosotomy (PC) has emerged as a potentially less morbid alternative that preserves prefrontal commissural connectivity. Detailed stereotaxic protocols for PC in rodent models are absent from the experimental literature.

Here we describe a detailed, step-by-step surgical protocol for both PC and TC in adult male Wistar rats (500–700 g; 8–9 months) using the WIN-PTZ kindling paradigm as the epilepsy induction model. The protocol encompasses: (1) a validated seven-session pentylenetetrazole (PTZ) kindling schedule (37.5 mg/kg, i.p., alternating days); (2) a complete multimodal anesthesia and analgesia regimen; (3) precise stereotaxic coordinates for both procedures, referenced from bregma using the Paxinos and Watson rat brain atlas; and (4) a postoperative PTZ rechallenge paradigm with behavioral outcome assessment using the revised Racine scale.

Protocol feasibility was confirmed in 11 animals with no intraoperative mortality and full postoperative tolerability. This protocol provides a feasible and repeatable experimental foundation for callosal disconnection research and can be adapted to chronic or focal epilepsy models with electrophysiological monitoring. Eight brains were available for histological analysis; Luxol Fast Blue myelin staining was attempted in a single specimen but proved non-diagnostic in this unoptimized preparation. On hematoxylin–eosin and toluidine blue sections, representative specimens showed morphological features of an in vivo callosal lesion (hemorrhage and reactive gliosis with a mononuclear infiltrate) within the transected territory, although prolonged fixation compromised tissue quality and precluded evaluation of the transection in many specimens. The protocol therefore supports feasibility, procedural repeatability, and preliminary morphological evidence of callosal disconnection, while confirmation of myelinated-fiber interruption at the tract level remains to be established.

## Highlights

• First detailed stereotaxic protocol for posterior corpus callosotomy in Wistar rats.
• Full anesthesia, stereotaxic coordinates, and postoperative monitoring provided.
• 0.5 mm lateral blade offset protects the superior sagittal sinus — key safety step.
• Executed in 11 rats with no intraoperative mortality, confirming feasibility.

## Graphical abstract

**Figure:**
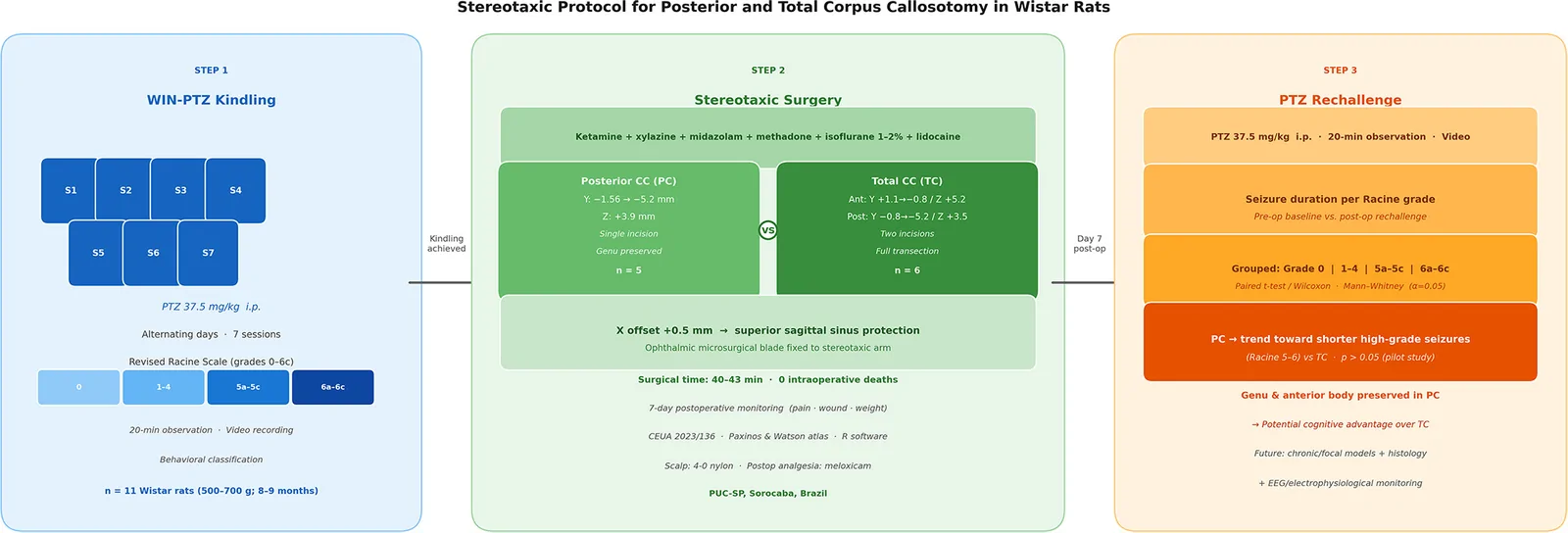

## Specifications table

**Table:** 

Subject	Neuroscience; Experimental Surgery
**Specific subject area**	Stereotaxic surgery; Epilepsy animal models; Corpus callosotomy; Surgical protocols
**Type of data**	Protocol description; Figures; Tables
**How data were acquired**	Stereotaxic surgical procedures in adult Wistar rats; PTZ kindling and behavioral observation; Video recording
**Data format**	Step-by-step protocol with stereotaxic coordinates, anesthesia doses, and behavioral classification criteria
**Parameters for data collection**	Adult male Wistar rats (500–700 g; 8–9 months); PTZ 37.5 mg/kg i.p.; Paxinos & Watson atlas; Revised Racine scale
**Description of data collection**	Animals underwent PTZ kindling (7 sessions) followed by stereotaxic callosotomy (PC or TC) and postoperative PTZ rechallenge at day 7
**Data source location**	**Institution:** PUC-SP — Faculdade de Ciências Médicas e da Saúde **City/Town/Region:** Sorocaba, São Paulo **Country:** Brazil
**Data accessibility**	The raw data underlying this protocol comprise: (i) video recordings of all PTZ kindling and postoperative rechallenge sessions; (ii) per-animal Racine-grade scoring sheets (duration of each seizure grade per session); (iii) surgical-duration logs; (iv) body-weight records (day of surgery and day of rechallenge); (v) postoperative pain-monitoring sheets; and (vi) the statistical analysis output (R). These files are not currently deposited in a public repository; they are retained by the corresponding author for at least 5 years following publication and are available on reasonable request.
**Related research article**	Cavalcanti VB et al. Comparative effects of posterior versus total corpus callosotomy in a PTZ-induced rat model of epilepsy: a methodological pilot study. *Experimental Brain Research* 2026, doi:10.1007/s00221-026-07375-x.

## Method details

### Animals

Fourteen adult male Wistar rats (*Rattus norvegicus*, Wistar strain; 500–700 g; 8–9 months old) were enrolled and entered the pentylenetetrazole (PTZ) kindling protocol. Three animals died during the kindling phase, before surgery: two during high-grade seizures and one from pneumonia (confirmed at necropsy). The remaining eleven animals underwent surgery and were allocated to posterior callosotomy (PC, n = 5) or total callosotomy (TC, n = 6); all eleven were included in the final analysis. Animals were obtained from the institutional vivarium of the Faculdade de Ciências Médicas e da Saúde, PUC-SP, Sorocaba, Brazil. All experimental procedures were conducted in compliance with national guidelines for the care and use of laboratory animals (CONCEA) and were approved by the institutional animal ethics committee (CEUA; protocol 2023/136).

Animals were housed in polyethylene cages lined with wood shavings (2–3 per cage) under controlled temperature (22 ± 1 °C), relative humidity (50–60%), and a 12 h light/dark cycle (lights on at 07:00 h). Standard pelleted chow and filtered water were provided ad libitum. Animals were individually identified by tail markings throughout the experiment. The advanced age and body-mass range used here (8–9 months; 500–700 g) were not a deliberate experimental design choice: the cohort aged beyond the originally planned window because acquisition of the controlled inhalational anesthetic (isoflurane) was delayed by national regulatory authorization, postponing surgery. This range is stated explicitly because the stereotaxic coordinates reported below were derived for it; users working with younger, lighter animals, with females, or with other strains should anticipate adjusting the coordinates to account for differences in skull dimensions, callosal anatomy, and pharmacological sensitivity.

### WIN-PTZ kindling protocol

**Fig. 1:**
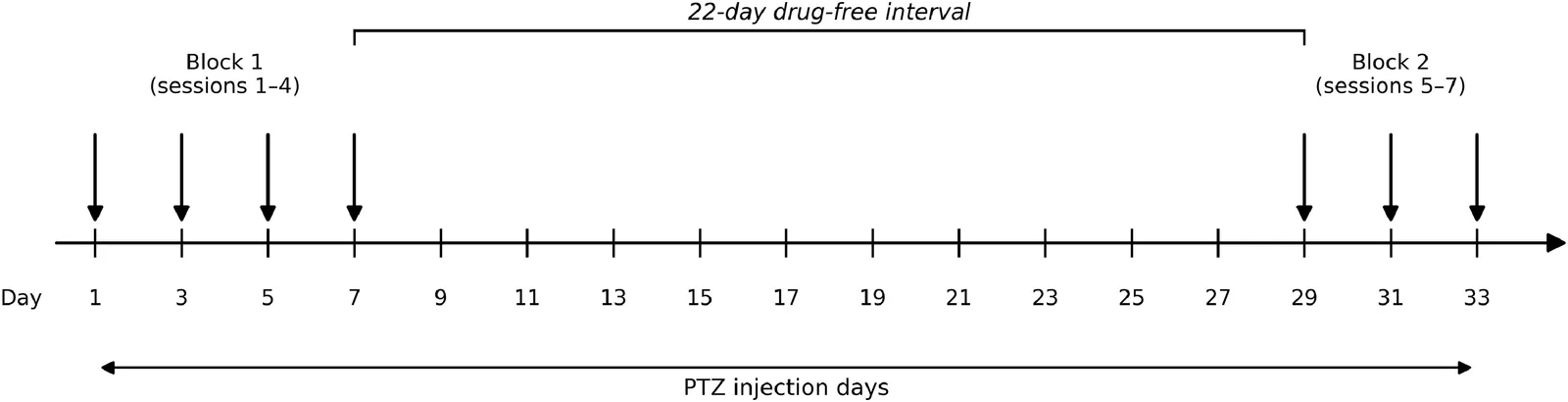
WIN-PTZ kindling protocol and surgical timeline. Seven PTZ sessions (37.5 mg/kg, i.p.) on alternating days (Block 1: sessions 1–4; 22-day drug-free interval; Block 2: sessions 5–7), followed by stereotaxic surgery and PTZ rechallenge at day 7 post-op. Each session involves a 20-min behavioral observation period with seizure classification using the revised Racine scale (grades 0–6c).

Epileptogenesis was induced using the window (WIN) variant of the pentylenetetrazole kindling method [[1], [2], [3], [4]]. PTZ (Sigma-Aldrich) was dissolved in 0.9% saline (stock concentration approximately 20 mg/mL) and administered by intraperitoneal (i.p.) injection at a dose of 37.5 mg/kg, adjusted to body weight, corresponding to an injection volume of approximately 1 mL per animal for the 500–700 g body-weight range. Injections were given on alternating days in two blocks: four injections on days 1, 3, 5, and 7 (Block 1; sessions 1–4), followed by a 22-day drug-free interval, and then three further injections on days 29, 31, and 33 (Block 2; sessions 5–7), totalling seven sessions (Fig. 1). This two-block schedule, with its intervening 22-day drug-free interval, is an intrinsic feature of the validated window paradigm [4] and not a study-specific modification. Each animal was weighed immediately before every session, and the 37.5 mg/kg dose was calculated from its current body weight; the solution was prepared fresh in 0.9% saline immediately before each injection, and the stock was kept refrigerated (4 °C) between sessions.

Each session consisted of a 20-minute behavioral observation period beginning immediately after injection, with continuous video recording. Seizure behavior was classified using the revised Racine scale for PTZ-induced seizures in rats [5] (Table 1). The preoperative behavioral baseline was calculated as the mean duration (seconds) of each Racine grade across the seven PTZ sessions. Sessions prematurely interrupted for humane reasons — specifically, prolonged high-grade seizures (≥ grade 5b) with signs of hypoxia (oral cyanosis) — were excluded from calculations. Interruption criteria were established *a priori* in consultation with the responsible veterinarian. This rule excluded only the individual interrupted session of the affected animal (not the whole animal) from that animal’s baseline, and was applied identically to the PC and TC groups. In total, five preoperative sessions in four animals were excluded: four sessions in three PC animals (IX-1, I-2 [two sessions], and II-2) and one session in one TC animal (I-3). Because interrupted sessions were among the most severe, their exclusion may bias the preoperative baseline toward a lower apparent seizure burden; this is acknowledged in the Limitations when interpreting the pre- versus postoperative comparison.

**Table 1:** Revised Racine scale for PTZ-induced seizures in rats [5].

Grade	Behavioral expression	Description
0	Whisker trembling	Facial twitching, vibrissae tremor
1	Behavioral arrest	Sudden immobility, staring
2	Facial clonus	Rhythmic facial and jaw movements
3	Neck spasms	Head nodding, neck muscle contractions
4	Clonic seizure (sitting)	Bilateral forelimb clonus, upright posture
5a	Clonic seizure (supine)	Forelimb clonus, loss of righting reflex
5b	Tonic-clonic (prone)	Generalized tonic-clonic in prone position
5c	Tonic seizure	Sustained tonic extension
6a	Clonic seizure (lateral)	Forelimb clonus in lateral decubitus
6b	Tonic-clonic (lateral)	Generalized tonic-clonic, lateral decubitus
6c	Wild jumping	Explosive jumping, loss of postural control

**Important note on seizure severity:** Although 37.5 mg/kg PTZ is conventionally described as a subconvulsive kindling dose, this protocol yielded high-grade seizures (≥ grade 5a) in a high proportion of sessions, likely reflecting the advanced age (8–9 months) of the animals. This proportion was calculated per completed PTZ session within each of the two kindling cohorts (denominator = total completed sessions in that cohort), yielding 60% of sessions in cohort 1 and 82% in cohort 2 (overall mean 71%; high-grade defined as ≥ grade 5a, consistent with the grouped Racine categories 5a–5c and 6a–6c used in the analysis). Researchers should anticipate this when planning monitoring and safety protocols.

### Anesthesia and analgesia

**Fig. 2:**
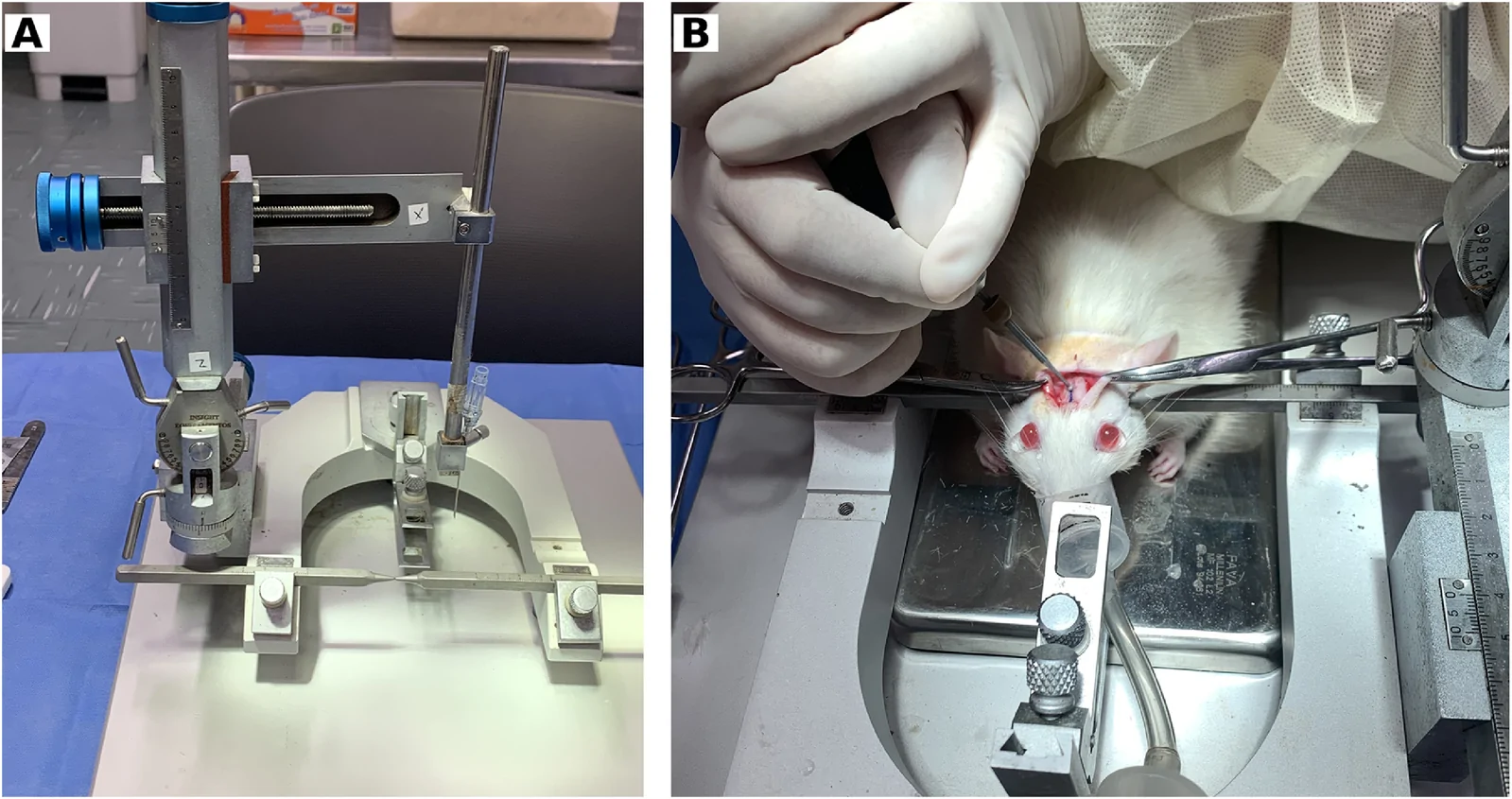
Stereotaxic apparatus used for callosotomy surgeries (Insight Instruments, Brazil). (A) Micromanipulator arm showing the anteroposterior, mediolateral, and dorsoventral (X, Y, Z) vernier scales and the ear-bar assembly used to set stereotaxic coordinates. (B) Animal positioned in the frame in the flat-skull configuration, secured by the ear bars and incisor/nose clamp during surgery. Original photographs by the authors.

All surgical procedures were performed under aseptic conditions using a stereotaxic frame (Fig. 2). Animals were anesthetized with an intraperitoneal combination of ketamine (100 mg/kg), xylazine (10 mg/kg), midazolam (5 mg/kg), and methadone (3 mg/kg). Anesthesia maintenance was achieved with inhaled isoflurane (1–2%) throughout the surgical procedure. This balanced, multimodal regimen combined the standard rodent surgical anesthetic pair — ketamine (dissociative) with xylazine (α2-agonist sedation and analgesia) [6] — with midazolam, which adds muscle relaxation and anticonvulsant cover appropriate to an epileptic cohort, and methadone for perioperative opioid analgesia; titratable inhaled isoflurane was used for maintenance. Anesthetic depth was confirmed before incision and monitored throughout by the absence of the pedal-withdrawal (nociceptive) reflex together with heart rate, respiratory rate, and respiratory pattern; maintenance isoflurane was titrated within the 1–2% range to keep a surgical plane (abolished pedal reflex with stable cardiorespiratory parameters). No adverse cardiorespiratory events were observed during the procedures.

Local analgesia was provided with subcutaneous lidocaine (7 mg/kg) at the incision site. Postoperative analgesia consisted of meloxicam (1 mg/kg, subcutaneously, once every 24 h), with the first dose given at the end of surgery and continued for up to three postoperative days, in accordance with the institutional NSAID standard operating procedure based on the UBC Animal Care Guidelines [6,7]. Rescue analgesia (an additional meloxicam dose) was available if pain indicators were present. Sterile saline was applied to the corneas to prevent desiccation. A thermoregulated heating pad was used to prevent hypothermia throughout surgery and recovery.

### Stereotaxic setup and surgical preparation

Animals underwent cranial trichotomy and antiseptic skin preparation. They were positioned in the stereotaxic frame in the flat-skull position, with the incisor bar adjusted to ensure the skull surface was level between bregma and lambda (±0.1 mm difference tolerated).

**Fig. 3:**
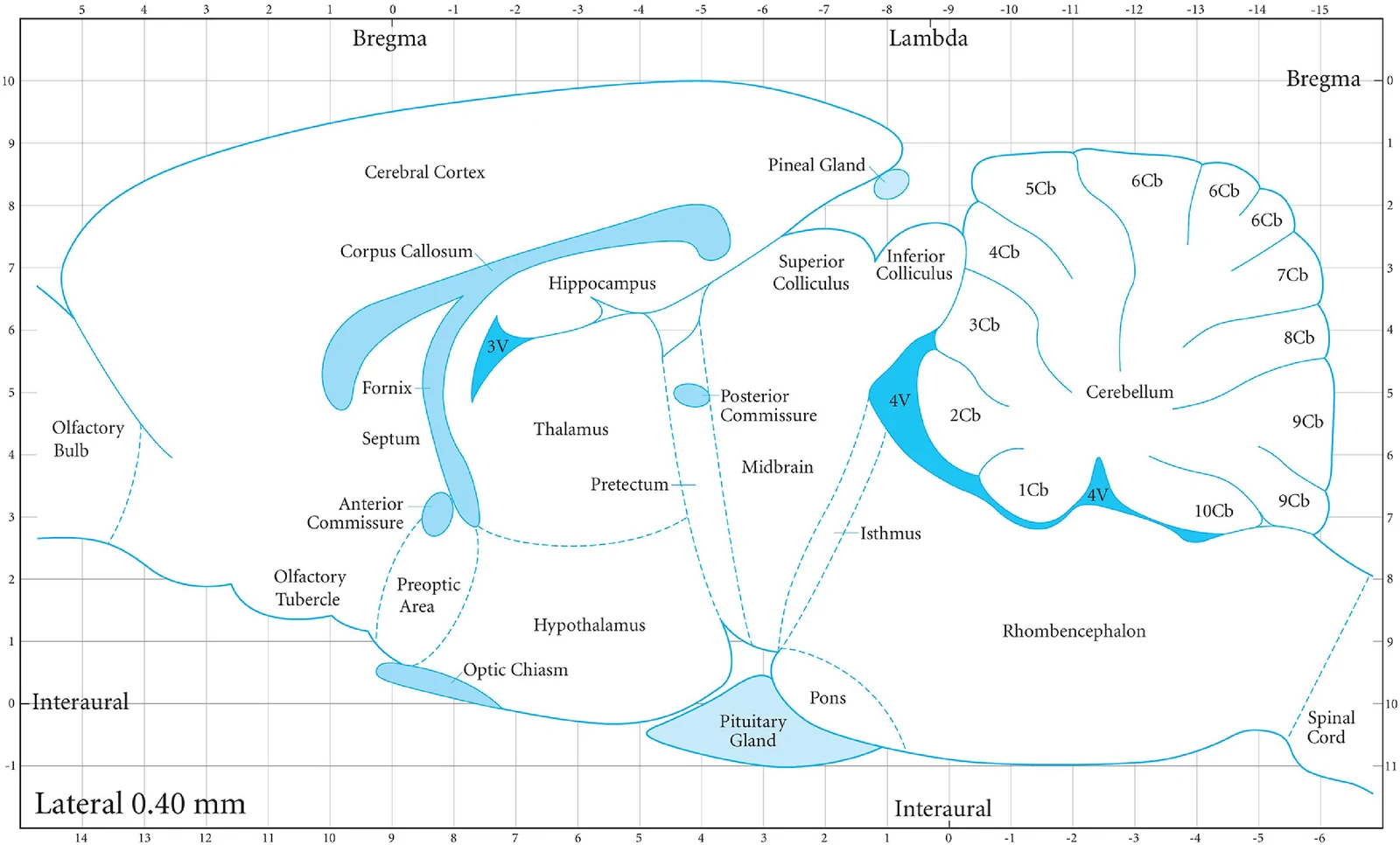
Sagittal reference section of the rat brain (Lateral 0.40 mm) from the Paxinos and Watson atlas, showing the corpus callosum and surrounding structures used to guide stereotaxic coordinate selection; bregma, lambda, and the interaural line are indicated as reference landmarks. Adapted from Paxinos G, Watson C. The Rat Brain in Stereotaxic Coordinates, 6th ed. Amsterdam: Elsevier; 2007.

Following a midline scalp incision and periosteal retraction, bregma was identified as the zero reference point (X = 0, Y = 0) for all stereotaxic coordinate calculations, following the Paxinos and Watson rat brain atlas [8] (Fig. 3); the callosal sectioning approach was adapted from the method of Crowne and Richardson [9]. All coordinates are referenced from bregma: anteroposterior (Y) and mediolateral (X) in the horizontal plane, and dorsoventral (Z) measured downward from the skull surface at the bregma reference point, with target depths taken from the atlas. A single craniotomy window was made over the planned incision using a saline-cooled dental bur, sized slightly larger than the longest planned incision (and therefore larger for total than for posterior callosotomy). Bone bleeding was controlled by saline irrigation alone (no bone wax), and the exposed cortex was kept moist with sterile saline. The dura was not separately opened or reflected; instead, the dura mater and underlying cortex were incised together by the microsurgical blade in a single pass along the full length of the planned trajectory (see below). Protection of the superior sagittal sinus relied on the 0.5 mm lateral offset rather than on any dural technique.

A disposable ophthalmic microsurgical blade (1.5 mm width) was mounted on the stereotaxic apparatus arm. A lateral offset of 0.5 mm from the sagittal midline (X = +0.5 mm) was applied to all incisions to avoid injury to the superior sagittal sinus. The blade’s cutting edge was oriented in the sagittal plane, aligned with the intended anteroposterior trajectory. It was advanced by the stereotaxic arm rather than freehand: lowered to the target Z coordinate and then advanced anteroposteriorly at constant depth in a single continuous pass that incised the dura and cortex together; one pass was used per incision. The offset was applied to the right of the midline (X = +0.5 mm) in all animals; the choice of side is arbitrary relative to the midline sinus, and an equivalent left-sided offset (X = −0.5 mm) would be expected to give the same result given the midline symmetry of the corpus callosum. Because the offset is small (0.5 mm), no asymmetry in transection extent is anticipated between sides.

### Posterior corpus callosotomy (PC)

**Fig. 4:**
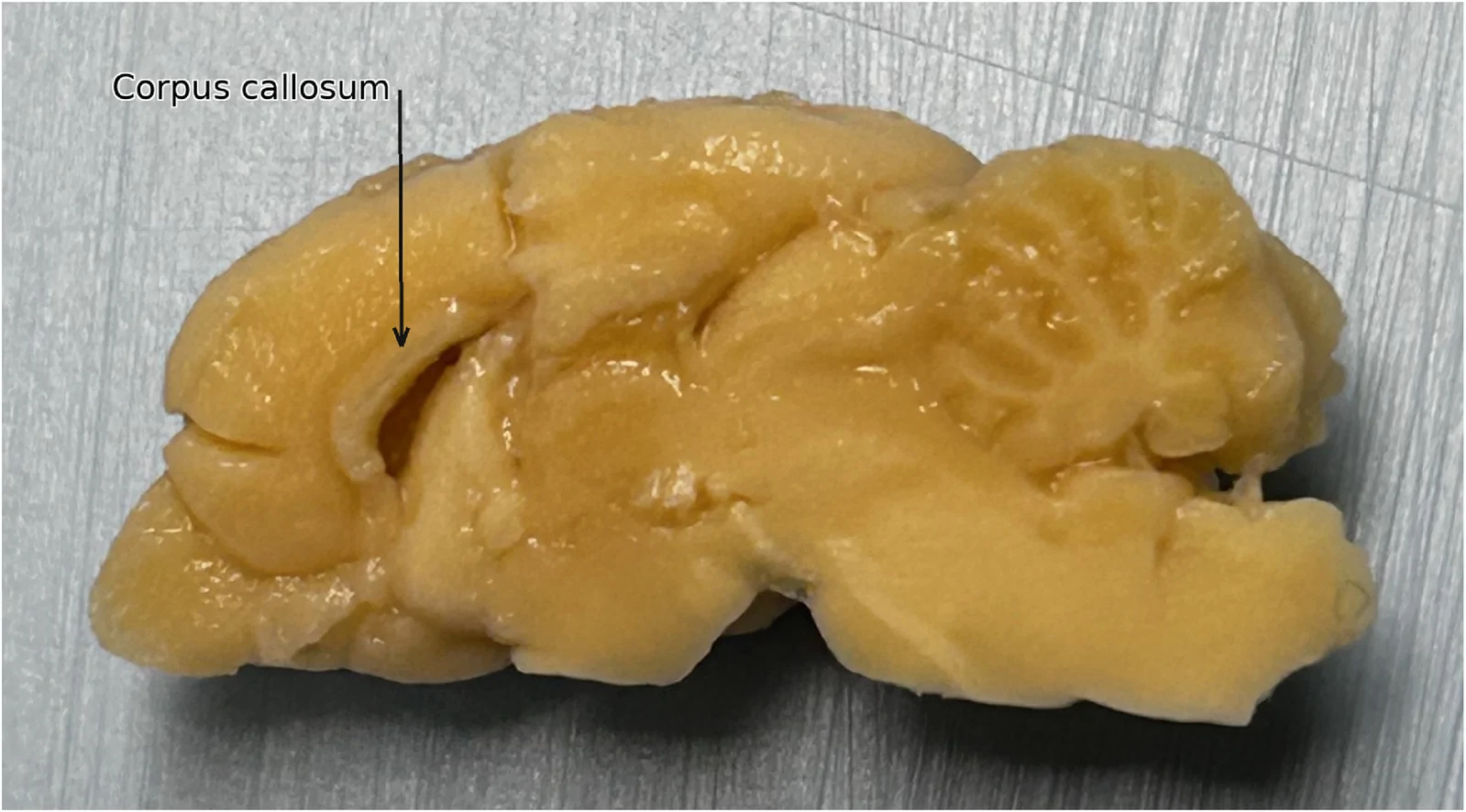
Macroscopic midsagittal view of a fixed adult Wistar rat brain, with the rostral pole to the left and the cerebellum to the right. The corpus callosum is indicated (arrow) as the pale, arched white-matter band coursing above the diencephalon. Original photograph by the authors.

Posterior callosotomy selectively transects the posterior body, isthmus, and splenium of the corpus callosum, preserving the genu and rostral body (Fig. 4). A single continuous incision was performed from anterior to posterior using the mounted ophthalmic blade.

Incision trajectory (referenced from bregma):

• **Start point:** X: +0.5 mm, Y: −1.56 mm, Z: +3.9 mm
• **End point:** X: +0.5 mm, Y: −5.2 mm, Z: +3.9 mm

The blade was lowered to the starting Z coordinate, advanced anteroposteriorly at constant depth, and withdrawn at the endpoint. Total incision length: 3.64 mm. The selected Z coordinate (3.9 mm from brain surface) corresponds to the dorsal surface of the posterior corpus callosum; the coordinate is intended to transect the dorsoventral extent of the callosal fibers at this plane while sparing the underlying hippocampal commissure and thalamus. Post-mortem macroscopic inspection of the callosum was carried out in all animals and was consistent with the intended lesion. On histological sections the callosal midline showed a discontinuity with a reactive tissue response at the expected level (see Histological Processing and Sectioning); myelin (Luxol Fast Blue) staining was attempted but was non-diagnostic following prolonged fixation, so this level is currently supported at the morphological rather than the myelin-tract level.

### Total corpus callosotomy (TC)

Total callosotomy achieves complete transection of the corpus callosum from genu to splenium. Due to the varying depth profile along the anteroposterior axis, two sequential incisions at different Z coordinates are required (Fig. 4). The two depth levels follow the dorsoventral profile of the callosum along the anteroposterior axis as read from the Paxinos and Watson atlas^8^: the genu and anterior body lie comparatively deep (blade entry to Z ≈ +5.2 mm), whereas the posterior body, isthmus, and splenium rise dorsally (entry to Z ≈ +3.5 mm), the corpus callosum spanning approximately AP +1.44 mm to AP −5.2 mm with a mean fiber thickness of about 0.4 mm. Coordinates relative to bregma were fixed across animals under flat-skull standardization and were not individually scaled to skull size or body weight (bregma was zeroed individually per animal, as is standard); users with markedly different skull dimensions should expect to rescale them.

**Anterior incision (genu and anterior body):**

• **Start:** X: +0.5 mm, Y: +1.1 mm, Z: +5.2 mm
• **End:** X: +0.5 mm, Y: −0.8 mm, Z: +5.2 mm

**Posterior incision (posterior body, isthmus, and splenium):**

• **Start:** X: +0.5 mm, Y: −0.8 mm, Z: +3.5 mm
• **End:** X: +0.5 mm, Y: −5.2 mm, Z: +3.5 mm

Together, the two incisions achieve complete callosal transection from genu (Y: +1.1 mm) to splenium (Y: −5.2 mm). The same lateral offset (X: +0.5 mm) was applied to both incisions. The anterior incision ends and the posterior incision begins at the same anteroposterior plane (Y = −0.8 mm); the two passes therefore meet at this coordinate, with the change in depth (Z = +5.2 → +3.5 mm) following the rising dorsal profile of the callosum. This shared transition plane is intended to ensure continuity of transection, with no intervening gap between the anterior and posterior segments.

### Hemostasis and wound closure

Hemostasis was maintained by gentle irrigation with sterile 0.9% saline throughout the procedure. Bleeding near the midline was additionally controlled by light compression with gauze; absorbable hemostatic material, cotton pledgets, and topical hemostatic agents were not used, and electrocautery was not used at any point in the procedure. The dura was not re-approximated. The scalp was closed with interrupted non-absorbable sutures (4–0 nylon). Animals were placed on a thermoregulated heating pad during recovery from anesthesia.

### Postoperative care and monitoring

Animals were returned to their home cages after recovery and monitored daily. Pain assessment was performed twice daily using the following behavioral indicators^6^:

• Vocalization upon handling
• Aggressive behavior or altered reactivity
• Anorexia or changes in feeding behavior
• Social withdrawal or absence of nest-building behavior
• Porphyrin ocular discharge (chromodacryorrhea)

Animals showing pain signs were treated with meloxicam per the institutional NSAID protocol [7]. Wound integrity was inspected daily; body weight was recorded on the day of surgery and at the time of PTZ rechallenge.

### Postoperative PTZ rechallenge

Seven days after surgery, all animals underwent a single PTZ rechallenge (37.5 mg/kg, i.p.), following identical procedures to the preoperative sessions: 20-minute observation window, continuous video recording, and seizure classification using the revised Racine scale [5]. For each animal, postoperative seizure duration per Racine grade was compared to the mean preoperative baseline.

### Histological processing and sectioning

Following the surgical and rechallenge procedures, brains were harvested for histological analysis. Eight of the eleven brains were available for histology, balanced across groups (PC: n = 4; TC: n = 4). Brains were immersion-fixed in 10% non-buffered formalin. Although the laboratory's standard fixation time is approximately one month, owing to logistical constraints in the interval between surgery and the histological analysis (undertaken only when revision was requested) these specimens remained in formalin, unprocessed, for a prolonged period of approximately six months prior to processing; this prolonged fixation is disclosed in full for transparency, although — as discussed in the staining and Limitations sections — it is an unlikely cause of the subsequent Luxol Fast Blue result. Tissue was processed in an automated tissue processor with a dwell time of one hour per station across twelve stations: three changes of formalin, a graded ethanol dehydration series (four changes at 85% and 95% followed by two changes of absolute ethanol, 99.5%), three changes of xylene for clearing, and two paraffin baths at 60 °C for impregnation. Specimens were embedded in paraffin at 60 °C and cooled. Serial coronal sections were cut on a rotary microtome at a nominal thickness of 5 µm, transferred on a flotation water bath (59–60 °C), and mounted on glass slides (54 × 60 mm). Three sections were retained per level for staining with hematoxylin–eosin, toluidine blue, and Luxol Fast Blue, as detailed below. General histological technique followed standard references [10].

Staining followed standard batteries. For hematoxylin–eosin, mounted sections were oven-dried (20 min), deparaffinized through five xylene baths (∼10 min each), and rehydrated through a descending ethanol series (two baths of absolute ethanol, one of 70%, and one of 50%, three dips each) to running water; sections were stained in hematoxylin for 15 min, rinsed in running tap water, differentiated in acid alcohol, rinsed again in running water (bluing), counterstained in eosin for 1 min, rinsed in running water, dehydrated through five baths of absolute ethanol, cleared, and coverslipped with Entellan (Merck). Toluidine blue staining was performed at approximately pH 4.5 (adjusted with acetic acid) for 1 min, followed by dehydration, clearing, and mounting. Luxol Fast Blue was applied following the Klüver–Barrera method^11^: sections were incubated overnight in a 60 °C oven, differentiated in lithium carbonate for approximately 30 s under microscopic control, counterstained with cresyl violet for 6 min, briefly rinsed in ethanol, cleared, and mounted.

**Fig. 5:**
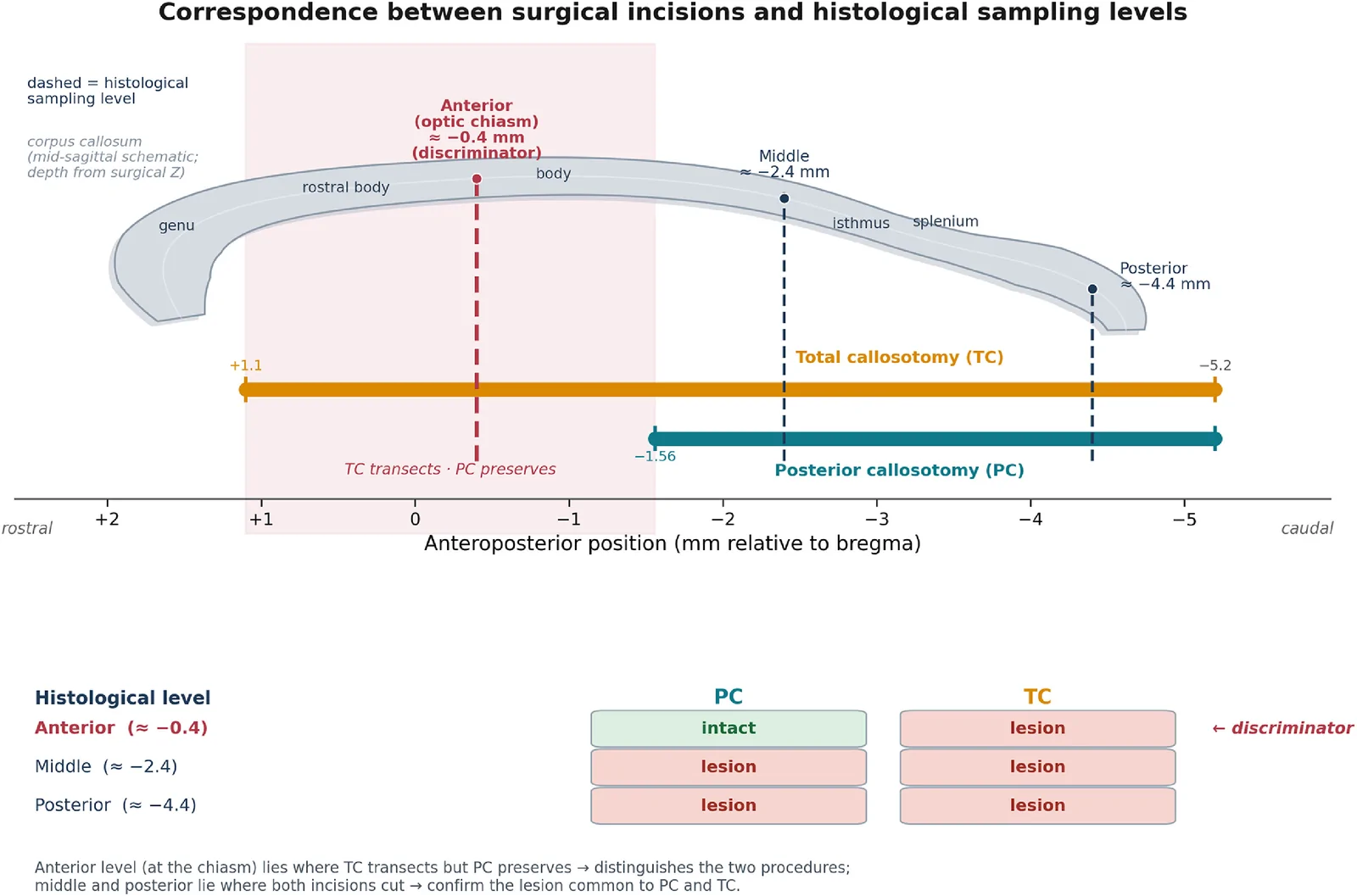
Correspondence between the surgical incisions and the histological sampling levels along the anteroposterior axis (mm from bregma; rostral positive). The corpus callosum is shown as a mid-sagittal schematic. Bars mark the extent of the total-callosotomy (TC; +1.1 to −5.2 mm) and posterior-callosotomy (PC; −1.56 to −5.2 mm) incisions; dashed lines mark the three histological levels relative to the optic chiasm (anterior ≈ −0.4, middle ≈ −2.4, posterior ≈ −4.4 mm). The shaded band (bregma +1.1 to −1.56 mm) is transected by TC but preserved by PC: the anterior level lies within it and distinguishes the two procedures (PC intact, TC lesion), whereas the middle and posterior levels lie where both incisions cut and document the shared lesion (table).

To sample the anteroposterior extent of the corpus callosum, three coronal levels were defined relative to the optic chiasm as a ventral internal landmark rather than to bregma, because bregma — the skull-suture reference used for the intraoperative coordinates — is not preserved in the extracted, fixed brain. The three levels were anterior (at the level of the optic chiasm), middle (approximately 2 mm caudal to the chiasm), and posterior (approximately 4 mm caudal). Because the optic chiasm spans approximately 0.8 mm rostrocaudally, its correspondence to skull-based coordinates is necessarily approximate; taking the centre of the chiasm at about bregma −0.4 mm in the Paxinos and Watson atlas [8], the anterior, middle, and posterior levels lie at approximately bregma −0.4, −2.4, and −4.4 mm, sampling the rostral body, the posterior body and isthmus, and the splenium of the corpus callosum, respectively. This scheme was designed so that the three levels together sample the callosal territory relevant to comparing the two procedures (Fig. 5). The anterior level lies within the segment that posterior callosotomy preserves — its incision beginning at bregma −1.56 mm — but that total callosotomy transects, its anterior incision beginning at bregma +1.1 mm; at this level the corpus callosum is therefore expected to remain intact after posterior callosotomy and to be transected after total callosotomy, providing the level at which the two procedures are expected to differ. The middle and posterior levels lie within the segment transected by both procedures and document the callosal lesion common to posterior and total callosotomy. Tissue rostral to the anterior level (the genu and rostral-most body) was not sampled histologically.

**Fig. 6:**
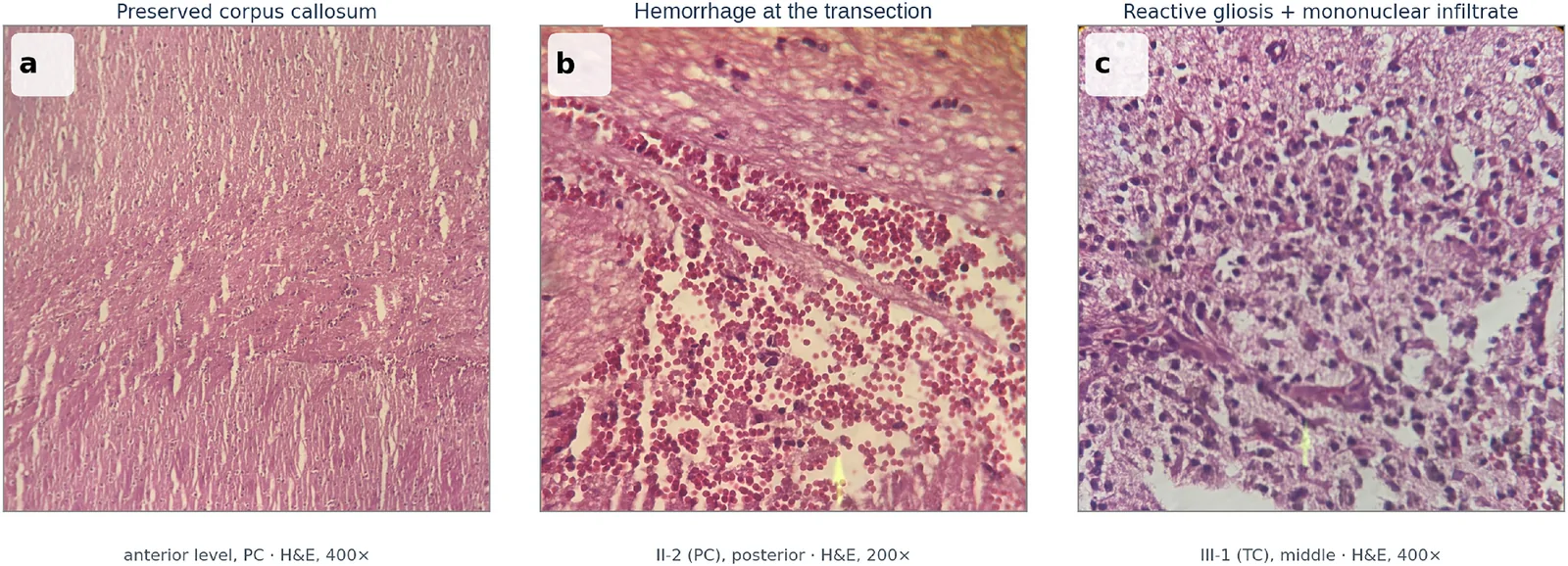
Representative histological findings supporting an in vivo callosal lesion. (a) Preserved, organized callosal white matter at the anterior level in a posterior-callosotomy specimen. (b) Hemorrhage at the callosal transection site (posterior level). (c) Reactive gliosis with a mononuclear inflammatory infiltrate at the lesion (middle level, total callosotomy). Hematoxylin–eosin; magnification as labelled. Tissue quality was limited by the prolonged fixation, and the panels are illustrative rather than quantitative.

General cytoarchitecture and lesion morphology were assessed on the hematoxylin–eosin and toluidine blue preparations. Luxol Fast Blue myelin staining [11] was attempted on a single specimen (I-1) but was non-diagnostic and was not pursued; because Luxol Fast Blue is reported to withstand very prolonged formalin fixation [12], its failure most likely reflects technical factors rather than the fixation interval, and callosal integrity was assessed at the morphological rather than the myelin-tract level. Tissue quality was, however, substantially compromised by the prolonged (approximately six-month) formalin fixation, and in many specimens the callosal midline could not be confidently evaluated for transection. Where the tissue was interpretable, representative specimens showed, within the transected territory (middle and posterior levels), morphological features of an in vivo lesion — hemorrhage at the callosal midline and reactive gliosis with a mononuclear inflammatory infiltrate — changes that reflect an in vivo process and therefore distinguish a surgically induced lesion from a post-processing sectioning artifact. At the anterior level, posterior-callosotomy material showed preserved, organized callosal white matter (Fig. 6). Because interpretation was limited and could not be applied systematically across animals and levels, these preparations are presented as supportive morphological evidence of an in vivo callosal lesion rather than as a quantitative confirmation of the anteroposterior extent of transection or a formal histological discrimination between the two procedures; definitive confirmation would require optimized myelin staining on tissue with a shorter fixation interval.

### Common pitfalls and troubleshooting

**Table:** 

Potential problem	Cause	Solution
Superior sagittal sinus injury	Blade positioned at true midline (X = 0)	Apply 0.5 mm lateral offset (X = +0.5 mm) to all incisions
Incomplete callosal transection	Incorrect Z depth or premature blade withdrawal	Verify Z coordinates from atlas; advance blade to target depth before lateral movement
Intraoperative hypothermia	Prolonged anesthesia duration	Use thermoregulated heating pad; monitor rectal temperature if surgery exceeds 60 min
Severe seizures at 'subconvulsive' PTZ dose	Advanced animal age increases seizure susceptibility	Define a priori interruption criteria (grade ≥ 5b + hypoxia signs); have emergency protocol ready

## Method validation

The protocol was successfully executed in 11 adult male Wistar rats (PC group: n = 5; TC group: n = 6). Protocol feasibility was confirmed by the following metrics:

**Surgical reproducibility:** Mean surgical duration was 45.4 ± 27.7 min for the PC group and 40.8 ± 12.3 min for the TC group (no significant difference; Wilcoxon test, p = 0.806) (Table 2).

**Table 2:** Surgical duration by surgical group.

Group	Mean ± SD (min)	Median [range] (min
Posterior callosotomy	45.4 ± 27.7	40 [25–93]
Total callosotomy	40.8 ± 12.3	41.5 [25–55]

No significant difference between groups (Wilcoxon test, p = 0.806).

**Baseline group equivalence:** No significant differences were found in body weight between the PC and TC groups at the time of surgery (*t*-test, p = 0.161), confirming baseline comparability.

**Kindling effectiveness:** PTZ kindling was successfully achieved in all 11 animals prior to surgery, with all animals displaying high-grade seizures (≥ grade 5a) during preoperative sessions, confirming adequate epileptogenesis.

**Intraoperative safety:** No intraoperative mortality occurred. All 11 animals survived the surgical procedure. The 0.5 mm lateral offset strategy successfully protected the superior sagittal sinus in all animals, with no vascular complications observed.

**Postoperative tolerability:** All 11 animals successfully completed the PTZ rechallenge seven days postoperatively.

## Limitations

The following limitations should be considered when applying this protocol:

• The WIN-PTZ kindling paradigm models chemically induced generalized epilepsy and does not reproduce focal epileptogenesis. The protocol may need adaptation for focal or chronic epilepsy models.
• The protocol was evaluated only in adult male Wistar rats (8–9 months). Results may differ in younger animals, in females, or other strains due to differences in skull thickness, callosal anatomy, and pharmacological sensitivity; the stereotaxic coordinates reported here should be regarded as a starting point requiring verification, and ideally pilot anatomical confirmation, in any different animal profile.
• The postoperative rechallenge was performed at 7 days post-surgery, capturing only acute effects. Longer follow-up periods with electrophysiological monitoring would be required to assess chronic or network-level effects of callosal disconnection.
• Myelin-stained (Luxol Fast Blue) confirmation of callosal transection was attempted but proved non-diagnostic, most likely for technical reasons: the stain was run on a single specimen without protocol optimization, and Luxol Fast Blue myelin contrast is highly sensitive to the differentiation step. Because Luxol Fast Blue staining is reported to withstand very prolonged formalin fixation, [12] the approximately six-month fixation interval is unlikely to be the primary cause. Lesion extent is therefore supported by intraoperative atlas-based targeting, post-mortem macroscopic inspection, and morphological histology (hematoxylin–eosin and toluidine blue), which showed a callosal discontinuity with a reactive tissue response but does not by itself establish myelinated-fiber interruption at the tract level. Definitive myelin-based confirmation would require re-staining additional sections with carefully titrated differentiation, ideally across at least one PC and one TC animal at the three anteroposterior levels; users reproducing the protocol are advised to include myelin-stained histological confirmation with particular attention to the differentiation step. In addition, the prolonged fixation compromised general tissue quality: in many specimens the callosal transection could not be confidently evaluated, so the morphological findings are supportive rather than systematic or quantitative.
• Sample size (n = 11) reflects the pilot nature of the study. Larger cohorts are recommended for definitive experimental comparisons.
• Sessions interrupted for humane reasons were excluded from the preoperative baseline. Because these were among the most severe seizures, their exclusion may bias the baseline toward a lower apparent seizure burden and conservatively attenuate the measured pre- versus postoperative difference; this should be considered when interpreting the rechallenge comparison.

## Related research article

Cavalcanti VB, Matsusako L, Canabarro L, Nakabashi F, Feitosa F, França WMG, Aguiar PHP. Comparative effects of posterior versus total corpus callosotomy in a PTZ-induced rat model of epilepsy: a methodological pilot study. Experimental Brain Research 2026, doi:10.1007/s00221-026-07375-x.

## Ethics statements

All animal experiments were performed in accordance with national guidelines for the care and use of laboratory animals (CONCEA — Conselho Nacional de Controle de Experimentação Animal) and were approved by the institutional animal ethics committee (CEUA, Faculdade de Ciências Médicas e da Saúde, PUC-SP; protocol number 2023/136). The sex of the animals is indicated: adult male Wistar rats were used exclusively in this study.

## Declaration of generative AI and AI-assisted technologies in the manuscript preparation process

During the preparation of this work the authors used Claude (Anthropic) to assist with figure/schematic preparation and formatting. After using this tool, the authors reviewed and edited the content as needed and take full responsibility for the content of the publication.

## CRediT authorship contribution statement

**Vitória Bernal Cavalcanti:** Conceptualization, Methodology, Investigation, Data curation, Writing – original draft, Writing – review & editing. **Larissa Matsusako:** Conceptualization, Methodology, Investigation, Writing – original draft, Writing – review & editing. **Luciana Canabarro:** Investigation, Data curation. **Fábio Nakabashi:** Investigation, Data curation. **Filipe Feitosa:** Investigation, Data curation. **Willy Marcus Gomes França:** Investigation, Data curation. **Paulo Henrique Pires de Aguiar:** Supervision, Project administration, Writing – review & editing.

## Declaration of competing interest

The authors declare that they have no known competing financial interests or personal relationships that could have appeared to influence the work reported in this paper.

## Data Availability

Data will be made available on request.
